# St. Bartholomew's Hospital

**Published:** 1904-01-30

**Authors:** Samuel Gee, Dyce Duckworth, T. Lauder Brunton, Norman Moore, Samuel West, John Langton, Harrison Cripps, W. Bruce Clarke, Anthony Bowlby, C. B. Lockwood, J. A. Ormerod, W. P. Herringham, H. H. Tooth, A. E. Garrod, J Calvert, F. H. Champneys, W. S. A. Griffith, D'Arcy Power, H. J. Waring, W. McAdam Eccles, R. C. Bailey, W. D. Harmer, W. H. H. Jessop, T. Holmes Spicer, A. E. Cumberbatch


### EDITOR'S LETTER-BOX.

**[Our Correspondents are reminded that prolixity is a great bar to publication, and that brevity of style and conciseness of statement greatly facilitate early insertion.]**

### ST. BARTHOLOMEW'S HOSPITAL.

*To the Editor of* "THE HOSPITAL."

SIR,—Since our former letter was published by you in December, 1903, several events have occurred which are of importance to those interested in this institution; we are therefore again asking you to give publicity to our views on the present situation.

We wish to emphasise the fact that the recent decision of the Governors to extend our site, either by acquiring more land or by utilising adjacent land, already in our possession, on which to erect the Nurses' Home and the Residential College for students meets with our unanimous approval and support.

It has been alleged that the plans for rebuilding are not sufficiently advanced to enable any accurate estimates to be made, and we should like therefore to say publicly that this is certainly not the case. The needs of the hospital for additional buildings were placed by us before our Governors more than two years ago, and the fullest details of even the floor space required by individual departments were also given. This has enabled the hospital architects to supply estimates of the cost of the required buildings, and the precise positions of the new blocks in a completed plan will not affect their conclusions.

It has always been the opinion of the Medical Council that all such additional buildings as they have asked for should be planned so as not to interfere with the rebuilding of the wards; and plans have now been submitted to us which support our views, and show that a completely new hospital can be satisfactorily erected on our site of six and a half acres. It will thus be seen that, instead of St. Bartholomew's Hospital having no sufficiently settled plans or estimates, the latter are in an advanced state. We are indeed quite convinced that more than enough time has been already spent in preliminary considerations, and we most heartily support the decision of the Governors to issue an appeal at once, for we know better than others can possibly know how urgent is the need for prompt action.

There is another matter that has also attracted attention to which we desire to allude, namely, the situation of St. Bartholomew's. Its great accessibility is a matter of the very highest importance to the poor of London. So long as the City is the heart of London and of the Empire, so long will access to it, and consequently to St. Bartholomew's, be cheap and easy, and so long will this charity remain a great consulting centre for the sick poor. The fact that its out-patients number about one hundred and fifty thousand in a year is a sufficient proof in itself of the usefulness of the hospital on its present site. There is no other district in any part of this city or in its suburbs where the hospital could be more advantageously placed, and none where a more healthy site could be found.

But the situation of St. Bartholomew's is further of importance to its medical school, and we may be pardoned for thinking that this is also a matter of material public interest. For many years past St. Bartholomew's has been not only one of the most important hospitals in England, but also the largest school of medicine. In this way the benefits of the Hospital are not restricted to its numerous patients, but are spread over Great Britain and its colonies. For the past twenty years an annual average of six hundred students have studied at St. Bartholomew's, and nearly one in every eight medical men in England can lay claim to the distinction of having learnt medicine and surgery there. The interests of the Hospital and of its school are one, and removal to some remote district would not only destroy the school, but would also by so doing completely alter the whole character of the institution itself.

Many other reasons might very easily be adduced in support of the appeal for sufficient funds both to maintain St. Bartholomew's in its present position and also to make it one of the most complete and modern of English hospitals of which the City of London might well be proud. But we have said enough to show the public that the present appeal is made in the best interests of the poor of London, and we know that many thousands of our fellow-citizens will now, and in after years, reap the benefits of those buildings which money alone can supply.

We are, Sir,

Yours faithfully,

| | |
|---|---|
| Samuel Gee | A. E. Garrod |
| Dyce Duckworth | J Calvert |
| T. Lauder Brunton | F. H. Champneys |
| Norman Moore | W. S. A. Griffith |
| Samuel West | D'Arcy Power |
| John Langton | H. J. Waring |
| Harrison Cripps | W. McAdam Eccles |
| W. Bruce Clarke | R. C. Bailey |
| Anthony Bowlby | W. D. Harmer |
| C. B. Lockwood | W. H. H. Jessop |
| J. A. Ormerod | T. Holmes Spicer |
| W. P. Herringham | A. E. Cumberbatch |
| H. H. Tooth. | |

